# OCT3/4 is a potential immunohistochemical biomarker for diagnosis and prognosis of primary intracranial germ cell tumors: a systematic review and meta-analysis

**DOI:** 10.3389/fnins.2023.1169179

**Published:** 2023-07-05

**Authors:** Yi Zhang, Mucong Li, Jifang Liu, Kan Deng, Huijuan Zhu, Lin Lu, Hui Pan, Renzhi Wang, Yong Yao

**Affiliations:** ^1^Department of Neurosurgery, Peking Union Medical College Hospital, Chinese Academy of Medical Science and Peking Union Medical College, Beijing, China; ^2^Department of Endocrinology, Peking Union Medical College Hospital, Chinese Academy of Medical Science and Peking Union Medical College, Beijing, China

**Keywords:** intracranial germ cell tumors (iGCTs), organic cation transporter 3/4 (OCT3/4), placental-like alkaline phosphatase (PLAP), immunohistochemical staining, diagnosis, prognosis

## Abstract

**Introduction:**

Intracranial germ cell tumors (iGCTs), comprising of germinoma (GE) and non-germinomatous GCT (NGGCT), are a group of heterogenous brain tumors. Immunohistochemical markers, such as placental-like alkaline phosphatase (PLAP), are commonly used in diagnosis but show moderate sensitivity. Organic cation transporter 3/4 (OCT3/4) has been proposed as a novel biomarker for diagnosis and prognosis of iGCTs. This paper aimed to compare OCT3/4 with PLAP as potential immunohistochemical biomarkers in iGCTs diagnosis and clarify the relationship between OCT3/4 and prognosis of patients with iGCTs.

**Methods:**

Meta-analyses were performed to estimate pooled percentage point differences in positive rates between OCT3/4 and PLAP, their sensitivities, and correlation between OCT3/4 and prognosis in iGCTs.

**Results:**

Nine articles were included representing of 241 patients. A fixed-effects model meta-analysis revealed that OCT3/4s positive rate was 8.6% higher (95% CI, 0.7% lower to 17.9% higher) than that of PLAP. Using fixed-effects models, sensitivities of OCT3/4 as a potential immunohistochemical biomarker in CNS GE and NGGCT were 85% (95% CI, 79% to 89%) and 56% (95% CI, 39% to 71%), respectively. In comparison, PLAP had lower sensitivities in both GE (73%; 95% CI, 64% to 91%) and NGGCT (43%; 95% CI, 27% to 61%). Moreover, OCT3/4 was significantly negatively correlated with 5-year progression free survival in patients with CNS GE (HR = 2.56, 95 % CI 1.47 to 4.44; *p* = 0.0008). Sensitivity analyses showed similar results.

**Discussion:**

This study provides the first comprehensive assessment of the efficacies of OCT3/4 and PLAP in iGCTs detection and prognosis prediction, indicating OCT3/4 seems to be a more sensitive and reliable immunohistochemical marker in iGCT diagnosis.

## 1. Introduction

Intracranial germ cell tumors (iGCTs) are a group of brain tumors with strong heterogeneity. The incidence of iGCTs is significantly higher in east Asian countries compared to western countries, accounting for approximately 2%–3% of primary intracranial neoplasm and 8%–15% of pediatric brain tumors (Jian et al., 2014). Mainly located in the midline region such as suprasellar compartment and pineal region, iGCTs are believed to be derived from primitive germ cells which aberrate into other sites during the development of gonads (Fetcko and Dey, 2018). Therefore, iGCTs recapitulate various stages of embryonal and extraembryonic development histologically. Germinoma (GE) and non-germinomatous GCT (NGGCT) are the two broad categories, whereas NGGCT is further subdivided into teratoma, yolk sac tumor, embryonal carcinoma, choriocarcinoma and mixed type (Zhou et al., 2019). GE is the most common type of iGCT. However, due to the limitation of sampling and obscuring background for histology, the diagnosis and differentiation of GE can be difficult. Also, the prognosis and treatment of GE remain controversial for lack of a stable prognostic molecular markers. Therefore, it is necessary to seek a helpful and meaningful biomarker to facilitate the diagnosis and predict the prognosis.

Earlier immunohistochemical markers for diagnosing GE include placental-like alkaline phosphatase (PLAP), C-KIT, and CD-117, but they only show moderate sensitivity and specificity, and pose no connection between the expression and prognosis. More recently, a novel stem cell marker, organic cation transporter 3/4 (OCT3/4, also called POU5F1), has been proposed as a more sensitive marker (Knuutinen et al., 2018). OCT3/4 is a transcription factor normally expressed in pluripotent stem and germ cells (Li et al., 2013). The role of OCT3/4 in the diagnosis, prognosis, and treatment of tumors have been discussed recently, including in testicular germ cell carcinoma, hepatocellular carcinoma, breast cancer, gastric cancer, pancreatic cancer and hypopharyngeal squamous cell carcinoma (Marinoff et al., 2017). Studies have demonstrated that OCT3/4 plays an important role in maintaining the stem cell state and initiating chemotherapy-resistance, thus correlating with worse prognosis in most cancer types.

However, the diagnostic and prognosis value of OCT3/4 in GE is not yet clear enough. Previous studies have reported the strong diffuse nuclear staining but variable expression among different patients of OCT4 as well. The meaning of such phenomena remains to be further elucidated. The meaning of such phenomena remains to be further elucidated. Thus, we carried out a meta-analysis to compare OCT3/4 with PLAP in iGCTs diagnosis and clarify the prognostic implication of OCT3/4 expression among patients with iGCTs.

## 2. Methods

### 2.1. Search strategy

A systematic literature search was conducted through databases of PubMed, Google Scholar, Embase, Wanfang, and CNKI before November 2022, with combined terms of OCT3/4 (“OCT3/4” or “organic cation transporter 3/4” or “octamer-binding transcription factor” or “POU5F1”), PLAP (“PLAP” or “placental alkaline phosphatase”), and GCT (“germinoma” or “germ cell tumors”). No language restriction was used in search strategy.

### 2.2. Study selection

Studies were considered eligible if: (1) Studies were original articles and from peer-reviewed journals including randomized clinical trials, cohort studies, cross-sectional studies, case–control studies, and case series; (2) The diagnosis of primary iGCT was confirmed by pathology, irrespective of any age, race, gender, clinical manifestation, living situation, tumor histology, site and grade; (3) The principle medical records were included in articles, including patient demographics and immunohistochemistry; (4) The study reported positive rates of immunohistochemical staining of OCT3/4 and/or PLAP in a population with iGCTs. Studies were excluded if: (1) Publication types were case reports, editorials, expert opinions, corresponding letters, commentaries, consensus statements, reviews, conference abstracts, or manuscripts under proceeding; (2) The study did not specify whether iGCT was a primary tumor; (3) The evaluation of OCT3/4 or PLAP was not performed. No minimal number of paired samples (that is, immunohistochemical staining results of OCT3/4 and PLAP were both available in the same patient) was set. For articles including duplicate patients or multiple publications of the same study results, only the most recently published with the largest combined samples were included. Two reviewers (Y Zhang and JF Liu) independently screened all eligible studies, and discordance was resolved by consensus.

### 2.3. Data extraction

Two reviewers (Y Zhang and MC Li) independently extracted the following data using a purpose-designed form: (1) Study characteristics: the first author’s name, study year, and country; (2) Population characteristics: number of all patients with iGCTs, number of patients with OCT3/4 available, number of patients with PLAP available, number of paired samples, mean age, and gender; (3) Histologic characteristics: histology types of iGCTs and corresponding numbers of patients, number of patients testing positive for OCT3/4, PLAP, and either OCT3/4 or PLAP; (4) Survival characteristics: mean or median follow-up years, overall relapse-free rates, and hazard ratios (HR). Mixed germ cell tumors (MGCTs) were defined as iGCTs containing at least two variable components from any subtypes of iGCTs. Semiquantitative grading of immunoreactivity (quantity score) was conducted as follows: 0, no tumor cells staining; 1+, >0% to 10% of tumor cells showing reactivity; 2+, >10% to 50% of tumor cells; 3+, >50% to 90% of tumor cells; 4+, >90% of tumor cells. Strongly positive immunoreactivity was defined as quantity score of more than 2+. HRs and standard errors were extracted directly from articles or calculation of figures or tables. Disagreements, if any, would be resolved by discussion of reviewers. To complete missing data, 3 original development papers were consulted to obtain missing information and 1 author replied.

### 2.4. Quality assessment

Risk of bias of the publications were assessed by the independent reviewers according to QUADAS-2 (Quality Assessment of Diagnostic Accuracy Studies 2) tool (Whiting et al., 2011). Patient selection, performance of the index test, performance of the reference test, and flow and timing were evaluated on this scale.

### 2.5. Data synthesis and analysis

Pathologic diagnosis of iGCT was considered as the gold standard through analyses. The primary aim of our study was the differences between positive rates of OCT3/4 and PLAP in immunohistochemical staining of CNS GE. Pooled percentage point differences and 95% confidence intervals (CIs) in sensitivity between OCT3/4 and PLAP were estimated with 6 out of 9 included studies. One of the secondary aims of our study was the sensitivities of OCT3/4 and PLAP for CNS GE and NGGCT detection. The specificity of OCT3/4 and PLAP for iGCTs detection could not be assessed due to the lack of non-iGCT cases in most of included studies. In addition, to evaluate the correlation between OCT3/4 and survival outcomes of CNS GE, we synthesized the time-to-event HRs as measure.

R (version 4.2.2, R Foundation) and RevMan software (version 5.4, Cochrane) were used for statistical analyses. Package meta (version 6.0-0) and metafor (version 3.8-1) in R were used to evaluate the percentage point differences and standard errors (SE) in sensitivity between OCT3/4 and PLAP for each study using the Wilson method (Newcombe, 1998). For calculating pooled estimates in both primary and secondary analyses, fixed-effects or random-effects meta-analysis was conducted using the inverse variance method with logit transformation when pooling and back transformation after pooling. Heterogeneity among the included studies was reported using the I^2^ statistic (Higgins et al., 2003). I^2^ value of less than 40%, 40%–75%, and more than 75% were regarded as unimportant, moderate, and considerable heterogeneity (Higgins et al., 2019). Fixed-effects models were used if there was unimportant heterogeneity among the studies, otherwise random-effects models were used (DerSimonian and Laird method; Dickersin, 1992). Sensitivity analyses with an individual study excluded at a time were conducted to evaluate the robustness of pooled results.

## 3. Results

### 3.1. Search results and characteristics of included studies

**Figure 1:**
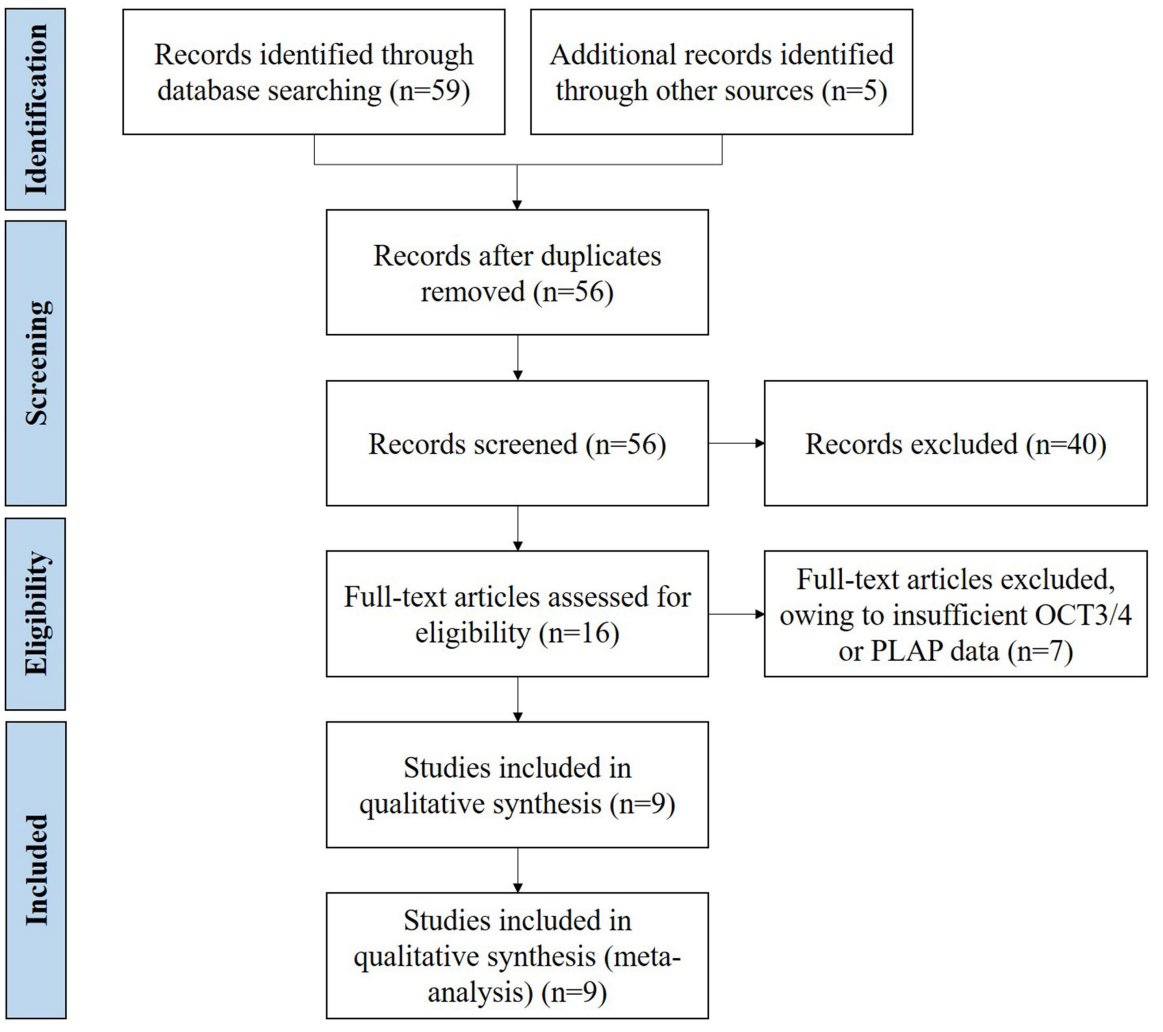
Literature search and selection.

A total of 64 records were identified (Figure 1). Of the relevant studies, 8 duplicate studies were removed, and 40 studies were excluded after title and abstract screening. Throughout full-text assessments were conducted for the rest 16 studies. Finally, 9 eligible studies with data on OCT3/4 and/or PLAP immunohistochemical staining results in iGCTs were included (Hattab et al., 2005; Teng et al., 2005; Santagata et al., 2006; Iczkowski et al., 2008; Ngan et al., 2008; Wanggou et al., 2012; Gao et al., 2014; Pantazis et al., 2014; Zhang et al., 2018). The general characteristics of included studies are summarized in Table 1. Overall, 9 studies conducted in China, United States, and Germany were included, comprising 241 participants with 239 (99.17%) immunohistochemical staining results of OCT3/4 (from 9 studies), 194 (80.50%) immunohistochemical staining results of PLAP (from 7 studies). We retrieved paired immunohistochemical staining results of OCT3/4 and PLAP in 111 GE (46.06%) from 5 studies. The mean age of included participants were 14.24 years and the male to female ratio was 1.66:1. Of 241 patients in these 9 included studies, GE accounted for 85.06% of all iGCTs. In view of NGGCT, teratoma (11, 30.56%) was the most common histologic type, followed by mixed germ cell tumor (9, 25.00%), yolk sac tumor (3, 8.33%), embryonal carcinoma (1, 2.78%), choriocarcinoma (1, 2.78%), and the rest 11 of NGGCT were with undefined histologic type.

**Table 1:** Summary characteristics of 9 included studies.

References	Country	Antibody (type/dilution/vendor)	Participants included (all patients with iGCTs/OCT3/4/PLAP/paired samples)	Mean age (years)	Male/female	Histology (no.)
Gao et al. (2014)	China	OCT3/4: Goat anti-IgG/ 1:50/Santa Cruz (c-20, sc-8,629); PLAP: Mouse anti-human/Working solution/DaKo (GM719102)	34/32/34/32	16.9	25/9	GE (23); T (7); MGCT (2); EC (1); CC (1)
Hattab et al. (2005)	United States	OCT3/4: Polyclonal goat anti-OCT3/4 antibody/1:500/Santa Cruz; PLAP: Polyclonal rabbit anti-PLAP antibody/Prediluted/Signet Pathology Systems, Inc., Dedham, MA (catalog no. 258–13)	25/25/25/25	NA	NA	GE (25)
Iczkowski et al. (2008)	United States	OCT3/4: Goat antibody/ 1:40/Santa Cruz; (c-20, sc-8,629); PLAP: NA/Prediluted/Ventana	30/30/30/0	NA	NA	GE (30)
Ngan et al. (2008)	China	OCT3/4: Anti-OCT3/4 antibody/1:250/Santa Cruz (c-20, sc-8,629); PLAP: Polyclonal/1:50/Dako	17/17/11/11	14.99	15/2	GE (5); MGCT (5); T (4); YST (3)
Pantazis et al. (2014)	Germany	OCT3/4: Mouse monoclonal anti-OCT3/4/1:500/Abcam; PLAP: Mouse monoclonal PLAP/1:25/Dako	23/23/21/21	16.35	18/5	GE (21); MGCT (2)
Santagata et al. (2006)	United States	OCT3/4: C-10/1:2000/Santa Cruz; PLAP: 8A9/1:250/Dako	12/12/12/12	13.92	8/4	GE (12)
Teng et al. (2005)	China	OCT3/4: Mouse-anti-human monoclonal anti-OCT3/4/1:100/Santa Cruz PLAP: Mouse-anti-human monoclonal anti-PLAP/Working solution/ZSGB-Bio	8/8/0/0	NA	NA	GE (8)
Wanggou et al. (2012)	China	OCT3/4: Polyclonal rabbit anti-OCT3/4 antibody/1:100/No. AB3209, Millipore	31/31/0/0	13.9	21/10	GE (31)
Zhang et al. (2018)	China	NA	61/61/61/61	12 (median)	24/37	GE (50); NGGCT (11)

iGCT, intracranial germ cell tumor; OCT3/4, octamer-binding transcription factor 3/4; PLAP, placental alkaline phosphatase; GE, germinoma; T, teratoma; YST, yolk sac tumor; EC: embryonal carcinoma; CC: choriocarcinoma; MGCT: mixed germ cell tumor; NA, not available.

### 3.2. Study quality

**Figure 2:**
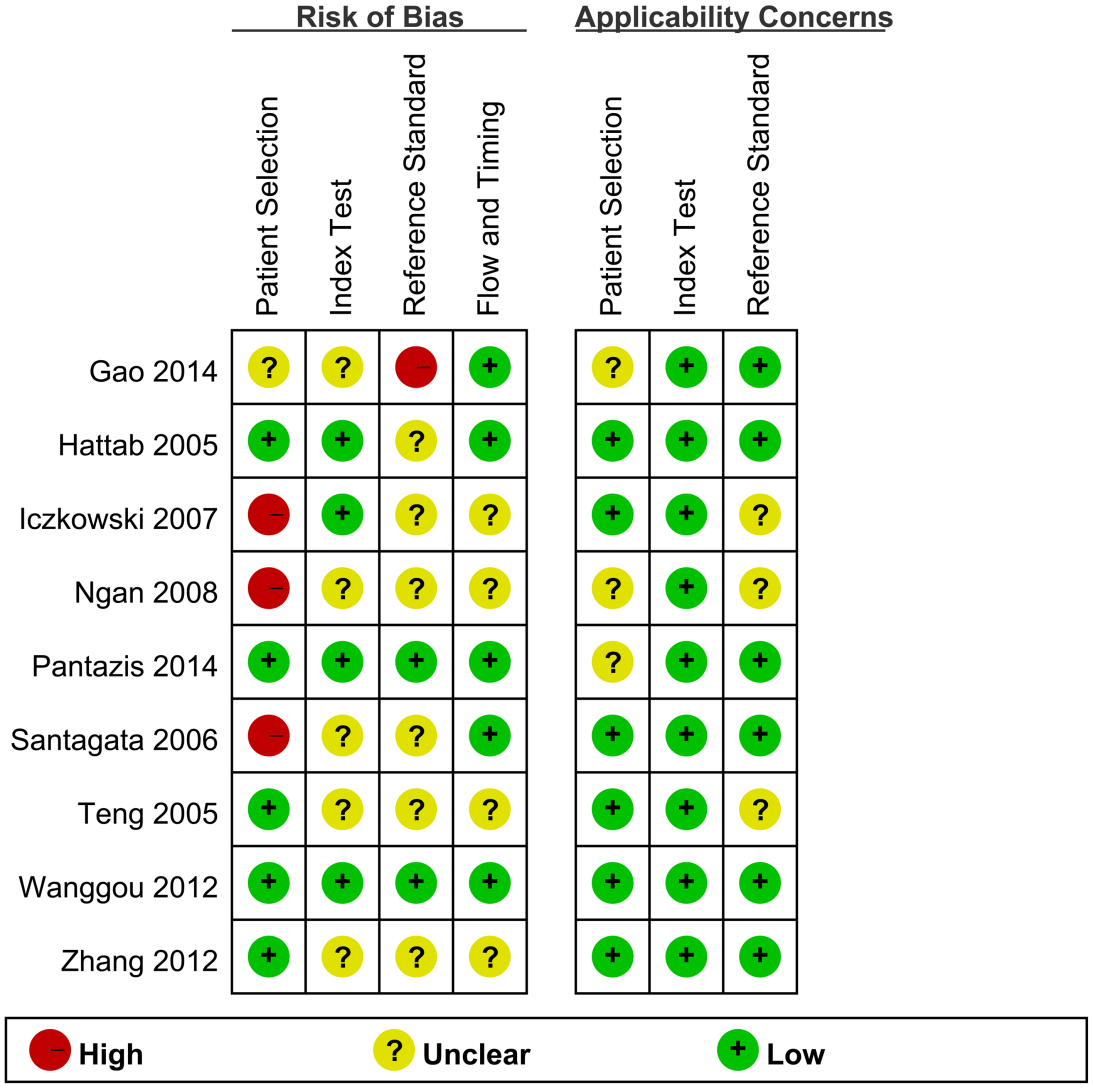
Quality assessment of 9 included studies.

Figure 2 presents results of quality scoring measured by QUADAS-2. Across all studies, 4 (44.44%) were at high or unclear risk of bias or applicability concerns in 4 or more domains. Seven (77.77%) of nine studies were at high or unclear risk due to the interpretation of reference standard results probably with the knowledge of the results of index tests, five (55.55%) of nine studies were at high or unclear risk due to the inappropriate interpretation of the results of index tests, four (44.44%) of nine studies were at high or unclear risk of patient selection bias, and four (44.44%) of nine studies were at high or unclear risk of flow and timing.

### 3.3. Percentage point differences in positive rates between OCT3/4 and PLAP in CNS GE

**Figure 3:**
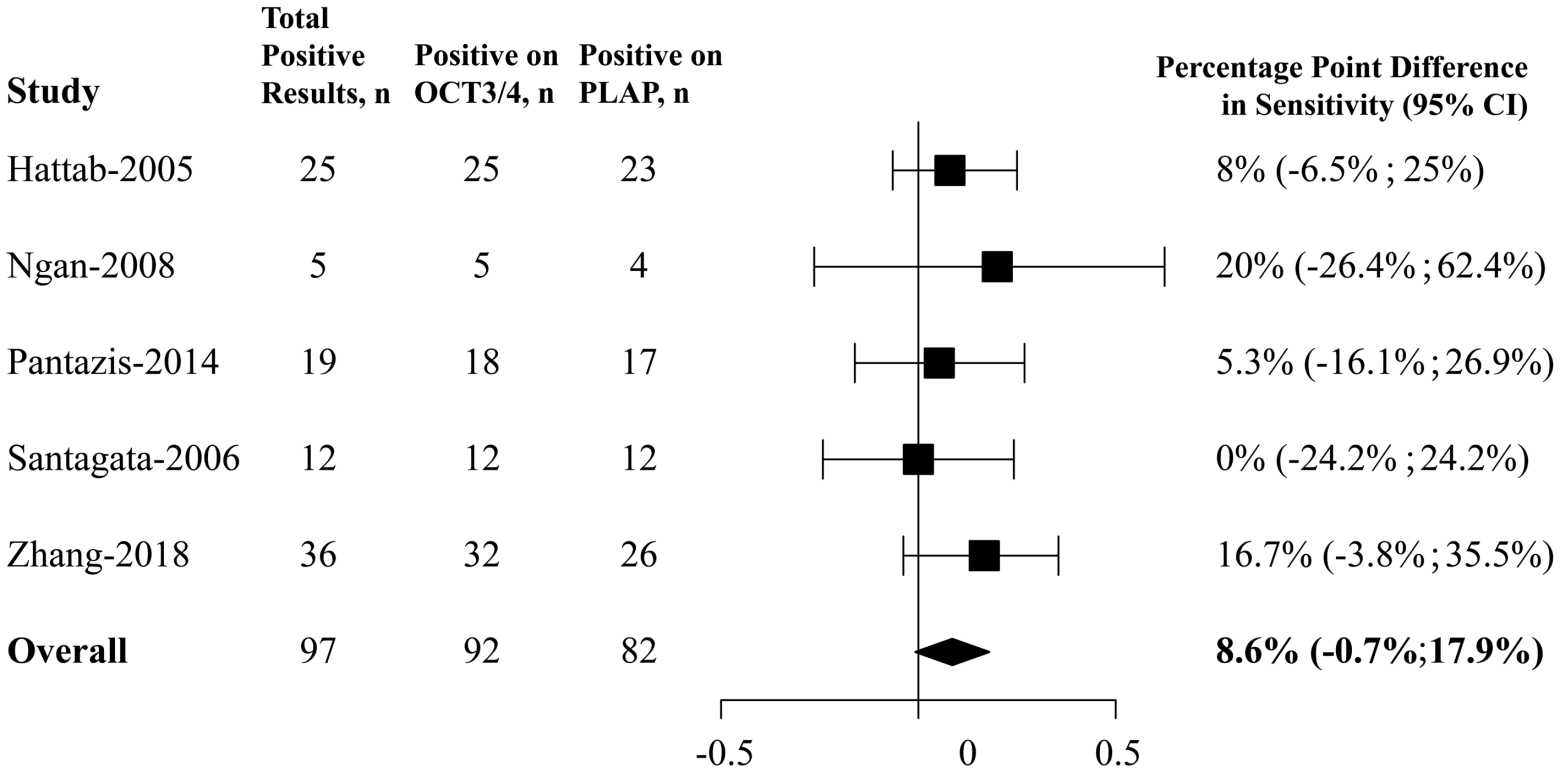
Forest plots of percentage point differences in positive rates between OCT3/4 and PLAP in CNS germinoma. CNS, central nervous system; OCT3/4, octamer-binding transcription factor 3/4; PLAP, placental alkaline phosphatase; CI, confidence interval.

As showed in Figure 3, among 111 paired samples of CNS GE included, 97 (87.39%) were positive on either OCT3/4 or PLAP. In our primary analysis, using a fixed effect model, it was estimated that OCT3/4’s positive rate was 8.6 percentage points higher (95% CI, 0.7 percentage points lower to 17.9 percentage points higher) than that of PLAP, suggesting that OCT3/4 probably had a better performance in detecting CNS GE, and thus OCT3/4 is recommended as a clinical biomarker for diagnosis. Heterogeneity based on the I^2^ statistic (0%) was unimportant.

### 3.4. Sensitivities of OCT3/4 and PLAP in CNS GE and NGGCT

**Figure 4:**
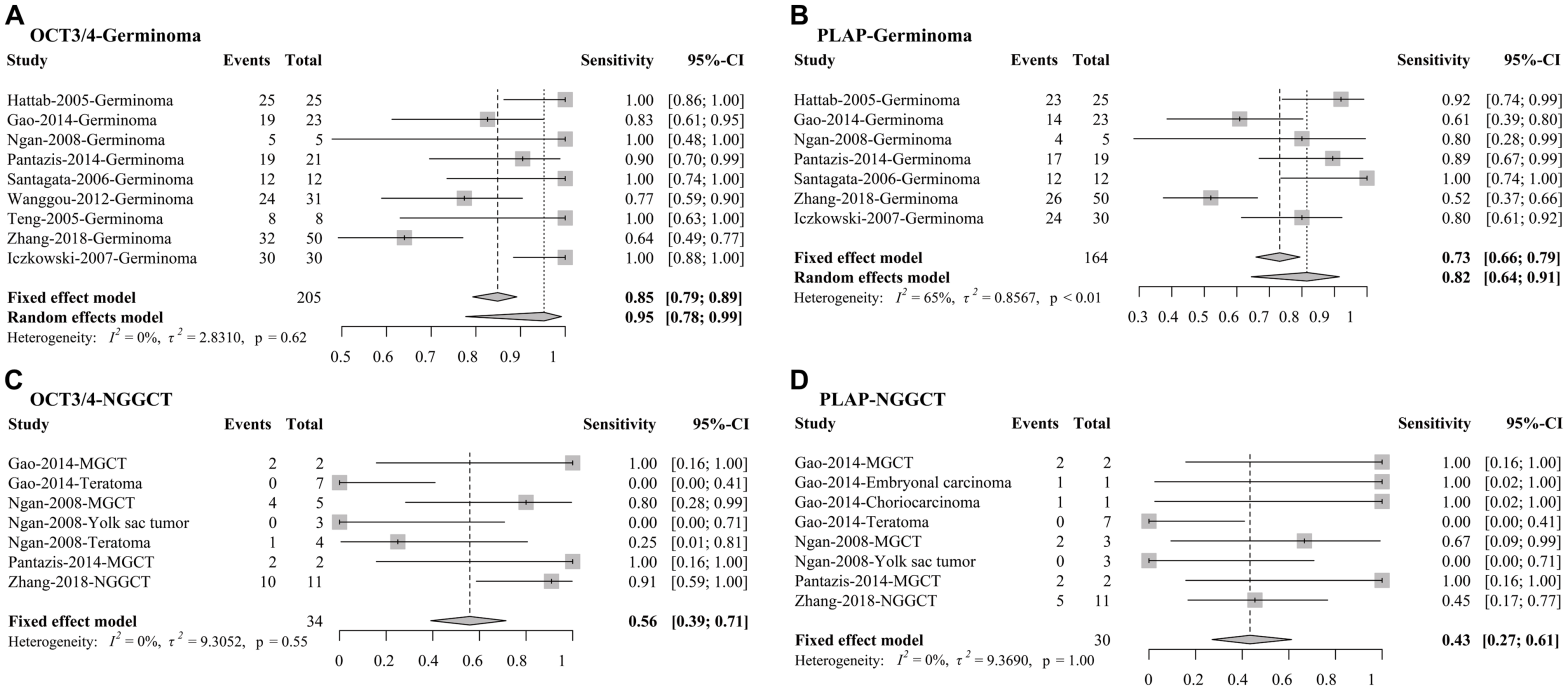
Forest plots of sensitivities of OCT3/4 and PLAP in **(A,B)** CNS germinoma and **(C,D)** NGGCT. CNS, central nervous system; OCT3/4, octamer-binding transcription factor 3/4; PLAP, placental alkaline phosphatase; CI, confidence interval.

For our secondary analysis, it was revealed that OCT3/4 had a pooled sensitivity of 85% (95% CI, 79% to 89%) among 205 CNS GE samples and 56% (95% CI, 39% to 71%) among 34 CNS NGGCT (Figure 4) using fixed-effects models. In comparison with OCT3/4, PLAP had lower sensitivities in detecting both CNS GE and NGGCT, with pooled sensitivities of 73% (95% CI, 64% to 91%) among 164 CNS GE and 43% (95% CI, 27% to 61%) among 30 CNS NGGCT using fixed-effects models. I^2^ indicated negligible heterogeneity in all models, except for the analysis of PLAP in GE (I^2^ = 65%), in which random-effects meta-analysis was conducted as well. For comparison, we also conducted random-effects meta-analysis of OCT3/4 in GE. OCT3/4 had a higher sensitivity (95%; 95% CI, 78% to 99%) than that of PLAP (82%; 95% CI, 64% to 91%) in random-effect models.

### 3.5. Survival outcomes of CNS GE patients with positive OCT3/4

**Figure 5:**
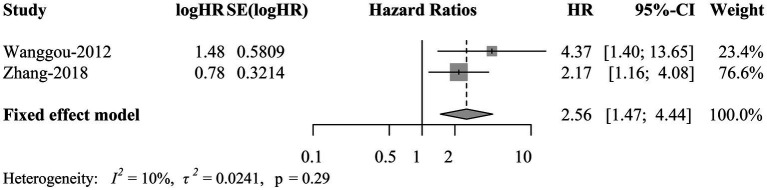
Forest plot of survival outcomes of CNS germinoma patients with positive OCT3/4. CNS, central nervous system; OCT3/4, octamer-binding transcription factor 3/4; TE; HR, hazard ratio; CI, confidence interval.

HRs and corresponding 95% CIs presenting survival outcomes of CNS GE patients with positive and negative OCT3/4 were collected in 2 studies. A fixed-effects meta-analysis showed that OCT3/4 was significantly negatively correlated with 5-year progression free survival (PFS) in patients with CNS GE (HR = 2.56, 95% CI 1.47 to 4.44; *p* = 0.0008; Figure 5). This result suggested that OCT3/4 is a promising predictor of the prognosis in CNS GE.

### 3.6. Sensitivity analysis and publication bias

Sensitivity analyses showed statistically robust results in the pooled percentage point differences in positive rates between OCT3/4 and PLAP in CNS GE (Supplementary Figure 1), and sensitivities of OCT3/4 and PLAP in CNS GE and NGGCT (Supplementary Figure 2).

## 4. Discussion

OCT3/4 is an 18-kDa POU-domain transcription factor encoded by the POU5F1 gene which is located on human chromosome 6 (6p21.31; Kobayashi et al., 2019). By binding to a specific octameric sequence motif, OCT3/4 is able to orchestrate a regulatory network in stem cell maintenance, including pluripotency, self-renewal and lineage commitment. For instance, it was demonstrated in ES cells that OCT3/4 controls the proliferation rate through an OCT4/Tcl1/Akt1 pathway (Clemente-Perivan et al., 2020). Meanwhile, OCT3/4 can also directly bind to the promoter region of Cyclin D1, together with miR302a-p16/p19-CDK4/6 pathway influenced, to control the G1/S phase of cell cycle. In normal human tissue, OCT3/4 is expressed during the early stage of embryo development and retains high expression in embryonic stem and germ cells (Kobayashi et al., 2019). The level of OCT3/4 is important to determination of stem cells fate. Researchers have found that high levels of OCT3/4 expression cause ESC differentiation into primitive endoderm and mesoderm, whereas low levels determine loss of pluripotency and differentiation toward trophectoderm. Together with OCT3/4, several other transcription factors (TFs), including SRY-box 2 (SOX2) and homeobox protein NANOG (NANOG) are also known to sit at the top of the regulatory hierarchy, controlling the stemness of ESCs by activating the self-renewal genes while suppressing the differentiation genes (Palma et al., 2013; Wang et al., 2019).

Due to its proliferative potential and self-renewal capacity, OCT3/4 has been linked to various aspects of cancer including tumorigenesis, invasiveness, metastasis, cancer cell stemness, drug-resistance and survival rate. In multiple cancer cells, researchers have identified thousands of OCT3/4 target genes involved in critical tumorigenesis pathway, such as PTEN signaling, TNC (metastasis) and MMP2(invasiveness). Together with NANOG and SOX2, OCT3/4 is widely regarded as marker of cancer stem cells, and higher expression of OCT3/4 has been proven to indicate worse clinical outcomes in most cancer types, i.e., more aggressive tumors, short overall survival and chemoresistance (Ling et al., 2012).

Germ cell tumors originate from primordial germ cells through neoplastic transformation, which pose a strong pluripotency, according to Teilum’s concepts. In intracranial germ cell tumors, GE is regarded as a prototype of all iGCTs, thus still remains its pluripotent potential, while each NGGCT arises from more differentiated stages of embryonic development starting from germ cells. Additionally, in some type of NGGCT like embryonic carcinoma, which is considered as neoplastic counterparts of ES cells, pluripotency of the tumor still exists (Khaiman et al., 2022). Therefore, as a marker of cell pluripotency, OCT3/4 is widely expressed in GE and serve with a strong diagnostic utility.

PLAP is a membrane-bound enzyme normally synthesized by placental syncytiotrophoblasts and released into maternal circulation after the 12th week of pregnancy (Borosky, 2014). PLAP is a traditional diagnostic marker for GE/seminoma in germ cell tumors. However, the fact that this marker lacks specificity and PLAP staining is located mainly on cell membrane and cytoplasm will cause causing difficulty in interpreting when the sample is mechanically crushed or frozen, especially under the circumstance that the tissue obtained is always small (Ichikawa et al., 2013). Compared with the cytoplasmic/membranous staining quality of PLAP, OCT4 is always highlighted in nuclei region of germinoma cells with little or no background. In the present study, OCT3/4 immunoreactivity is marginally significantly higher than PLAP and thus serves as a better diagnostic marker in sensitivity.

Although CNS GE displayed strong diffuse nuclear staining of OCT3/4 in at least some regions of the tumor, such expression was variable. In Wanggou’s study, OCT3/4 expression on tumor cells not only varies from case to case, but also from region to region in even the same section. Our study has summarized that higher OCT3/4 expression is correlated with worse prognosis of GE. This is not surprising as OCT3/4 has already been proved to play a critical role in cancer cell stemness maintenance thereby inducing a strong radiochemo-resistance. Our findings are of great clinical importance as OCT3/4 can not only assist in iGCT diagnosis but predict the patients’ outcome as well.

Cerebrospinal fluid (CSF) provides another highly sensitive diagnostic window when tumor biopsy is not applicable in iGCTs. Currently the CSF level of tumor markers AFP and β-hCG has been a routine examination for differential diagnosis and prognosis prediction (Sathitsamitphong et al., 2022). And studies have also demonstrated an increased level of s-kit (soluble isoform of c-kit) and PLAP in GEs, which suggest the possibility of establishing iCGT diagnosis based on CSF secreting patterns. However, the CSF level of OCT3/4 has not been available so far. And further study is required to incorporate OCT3/4 to establish a CSF proteomics panel. Besides, previous study has present cases of utilizing OCT3/4 in cerebrospinal fluid cytology diagnosis. Finding of OCT3/4 positive tumor cells in CSF indicates the seeding of original tumor sites into CSF and signifies the necessity of chemotherapy. Therefore, larger sample-sized studies are expected to expand the utility of OCT3/4 in CSF or other sites in the future.

In summary, OCT3/4 is a highly useful immunohistochemical marker in iGCT diagnosis. Moreover, our study has demonstrated the significance of OCT3/4 in prognosis of iGCT patients such as GE.

## Data availability statement

The original contributions presented in the study are included in the article/Supplementary material, further inquiries can be directed to the corresponding author.

## Author contributions

HP, RW, and YY: conception and design and studies evaluation. YZ, ML, JL, and KD: literature search. YZ, ML, JL, and KD: data extraction. YZ and ML: data analysis and manuscript drafting. KD, HZ, and LL: editing and revising the manuscript. All authors contributed to the article and approved the submitted version.

## Funding

This study was supported by National High Level Hospital Clinical Research Funding (no. 2022-PUMCH-B-114) from YY and Youth Science Foundation of Peking Union Medical College Hospital (no. PUMCH201911867) from YZ.

## Conflict of interest

The authors declare that the research was conducted in the absence of any commercial or financial relationships that could be construed as a potential conflict of interest.

## Publisher’s note

All claims expressed in this article are solely those of the authors and do not necessarily represent those of their affiliated organizations, or those of the publisher, the editors and the reviewers. Any product that may be evaluated in this article, or claim that may be made by its manufacturer, is not guaranteed or endorsed by the publisher.
