# Supplementary material for: OCT3/4 is a potential immunohistochemical biomarker for diagnosis and prognosis of primary intracranial germ cell tumors: a systematic review and meta-analysis
Source: Front Neurosci. 2023 Jul 5;17:1169179. doi: 10.3389/fnins.2023.1169179 (PMC10354551; doi:10.3389/fnins.2023.1169179)
Supplement: Supplementary file 1 [file Data_Sheet_1.DOCX]

Supplementary Material

OCT3/4 is a potential biomarker for diagnosis and prognosis of primary intracranial germ cell tumors: a systematic review and meta-analysis

Yi Zhang^1†^, Mucong Li^1†^, Jifang Liu^1^, Kan Deng^1^, Huijuan Zhu^2^, Lin Lu^2^, Hui Pan^2^, Renzhi Wang^1^, Yong Yao^1^

*** Correspondence:** Yong Yao, tigerfreeyy@126.com

# Supplementary Figures


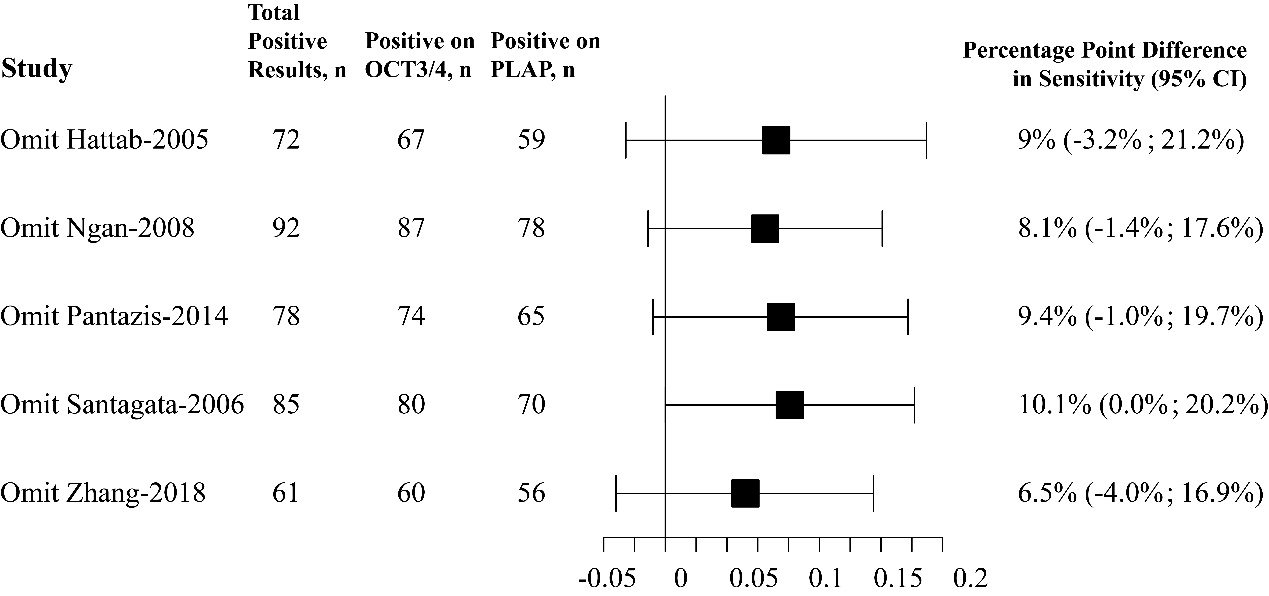


**Supple Fig. 1** Sensitivity analysis of percentage point differences in positive rates between OCT3/4 and PLAP in CNS germinoma. CNS, central nervous system; OCT3/4, octamer-binding transcription factor 3/4; PLAP, placental alkaline phosphatase; CI, confidence interval.


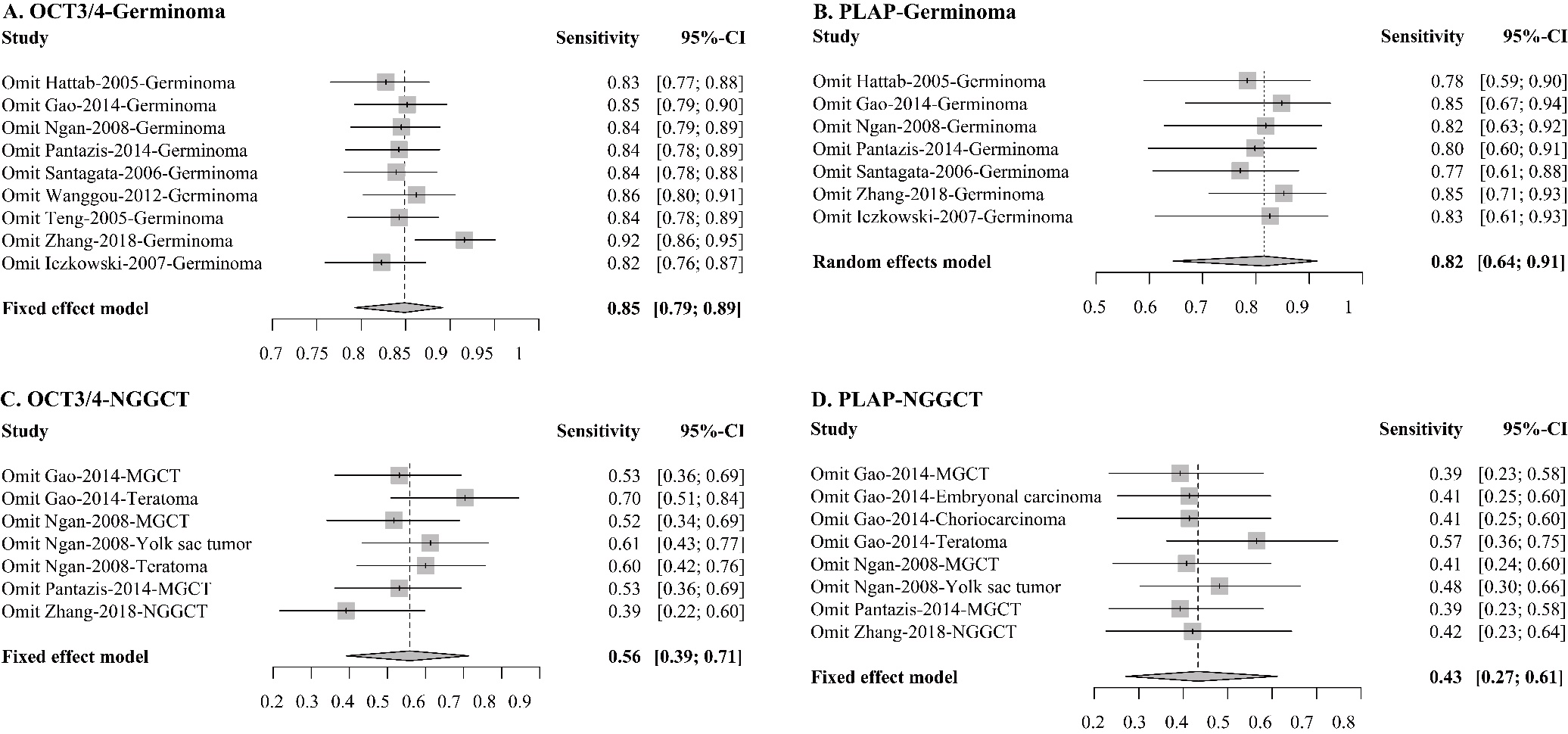


**Supple Fig. 2** Sensitivity analysis of sensitivities of OCT3/4 and PLAP in (A, B) CNS germinoma and (C, D) NGGCT. CNS, central nervous system; OCT3/4, octamer-binding transcription factor 3/4; PLAP, placental alkaline phosphatase; CI, confidence interval.
